# Case Report: Endovascular technique for intravascular foreign body retrieval in severely preterm neonate

**DOI:** 10.12688/f1000research.142275.1

**Published:** 2024-01-11

**Authors:** Marsha Ruthy Darmawan, Djuwita Adi Wahyono, Taufiq Fredrik Pasiak, Listyo Julia, Irhamni Irhamni, Terawan Agus Putranto

**Affiliations:** 1 Department of Radiology, Gatot Soebroto Central Army Hospital, Jakarta Pusat, DKI Jakarta, 10430, Indonesia; 2 Department of Radiology, Universitas Pembangunan Nasional Veteran Jakarta, Jakarta, DKI Jakarta, 10430, Indonesia; 3 Department of Anesthesiology, Gatot Soebroto Central Army Hospital, Jakarta Pusat, DKI Jakarta, 10430, Indonesia; 4 Department of Pediatric Surgery, Gatot Soebroto Central Army Hospital, Jakarta Pusat, DKI Jakarta, 10430, Indonesia

**Keywords:** Endovascular, interventional radiology, intravascular foreign body, preterm, neonate

## Abstract

**Background:**

Intravascular foreign bodies (IFB) in severely preterm neonates present critical clinical challenges. Traditional surgical removals are often risky and may not be feasible in these patients. This case report aims to detail a successful endovascular approach for IFB retrieval in a severely preterm neonate.

**Case:**

This is a case report of a 14-day old, male 30-week gestation neonate with a retained umbilical catheter fragment. The baby was referred after an unsuccessful explorative laparotomy by a paediatric surgery team. The endovascular procedure was performed using a 4 French (Fr) vertebra catheter and a One Snare wire in the right femoral region under low-dose fluoroscopy. The IFB was successfully removed. Post-procedure, the patient’s leukocyte count normalized, and he transitioned from ventilator support to a nasal cannula and eventually to room air within three days. The neonate could also tolerate oral intake, signalling a rapid recovery and low morbidity.

**Conclusions:**

This case report highlights the potential of endovascular approaches for IFB retrieval as viable alternatives to traditional surgeries, especially in neonates where surgical options are limited or have failed. Further research is needed to standardize such minimally invasive techniques in neonatal care.

## Introduction

Intravascular foreign bodies (IFB) in severely preterm neonates present a significant clinical challenge fraught with multiple risks including infection, vascular injury, and even death. Such events are exceedingly rare yet demand immediate attention given the vulnerable nature of the neonatal patient population. ^1^ Traditional surgical approaches for foreign body removal are not only risky but may also not be feasible due to the extremely delicate anatomical structures and limited physiologic reserves of preterm neonates. Therefore, safer and minimally invasive interventions are often preferred. ^2^

Endovascular techniques have gained prominence in adult and paediatric populations for various applications, including foreign body retrieval, because of their less invasive nature and shorter recovery time. However, the literature describing the efficacy, safety, and technical aspects of endovascular interventions for foreign body retrieval in severely preterm neonates is sparse. ^2^^–^^4^

This case report aims to detail a successful endovascular approach for intravascular foreign body retrieval in a severely preterm neonate. By doing so, we hope to contribute valuable information to the limited pool of knowledge on this subject and offer clinicians an alternative approach that could potentially minimize surgical risks while ensuring effective treatment. This case report is reported according to the CARE (case report guidelines) 2017 criteria. ^5^

## Case report

A 14-day old, Asian, male preterm neonate was referred to Gatot Soebroto Army Hospital, Indonesia due to intravascular foreign body. Previously, the neonate was born during his 30 weeks of gestation with birth weight of only 1,600 grams and birth height of 39 cm. He was then admitted to the neonatal intensive care unit (NICU) due to respiratory distress. During his admission, an umbilical catheter was placed for vascular access, and he was intubated. A week following the birth, his condition had improved, he was extubated and had continuous positive air pressure (CPAP) worn. However, a fragment of the umbilical catheter was left behind. Two intravenous antibiotics (ampicillin and gentamicin) were administered to the patient for over a week; however, his condition did not improve. During the physical examination, his respiratory rate was 55 times a minute with heart rate of 168 times per minute and peripheral oxygen saturation of 96% on ventilator.

**Figure 1.**
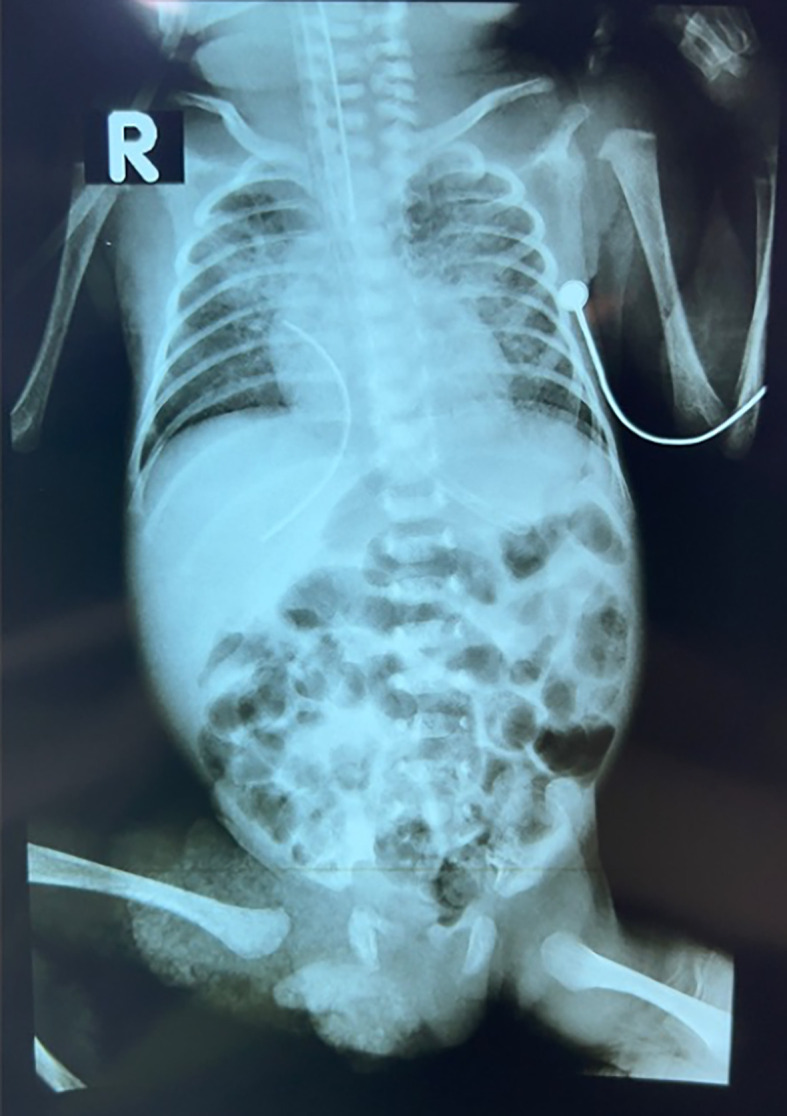
The preliminary thoracoabdominal X-ray image showing a radiopaque catheter at the right paravertebral region.

His laboratory results were significant for leucocytosis with normal thrombocyte and C-reactive protein. The other laboratory results were unremarkable. Based on the thoracoabdominal X-ray imaging, a radiopaque catheter was found at the right paravertebral region, behind the liver, at the level of Th8-L2 and pulmonary infiltrate ( Figure 1).

Following admission, the patient underwent explorative laparotomy by the paediatric surgeon team in order to extract the foreign body but to no avail. He was then referred to the interventional radiology department for intravascular foreign body extraction using endovascular approach.

**Figure 2.**
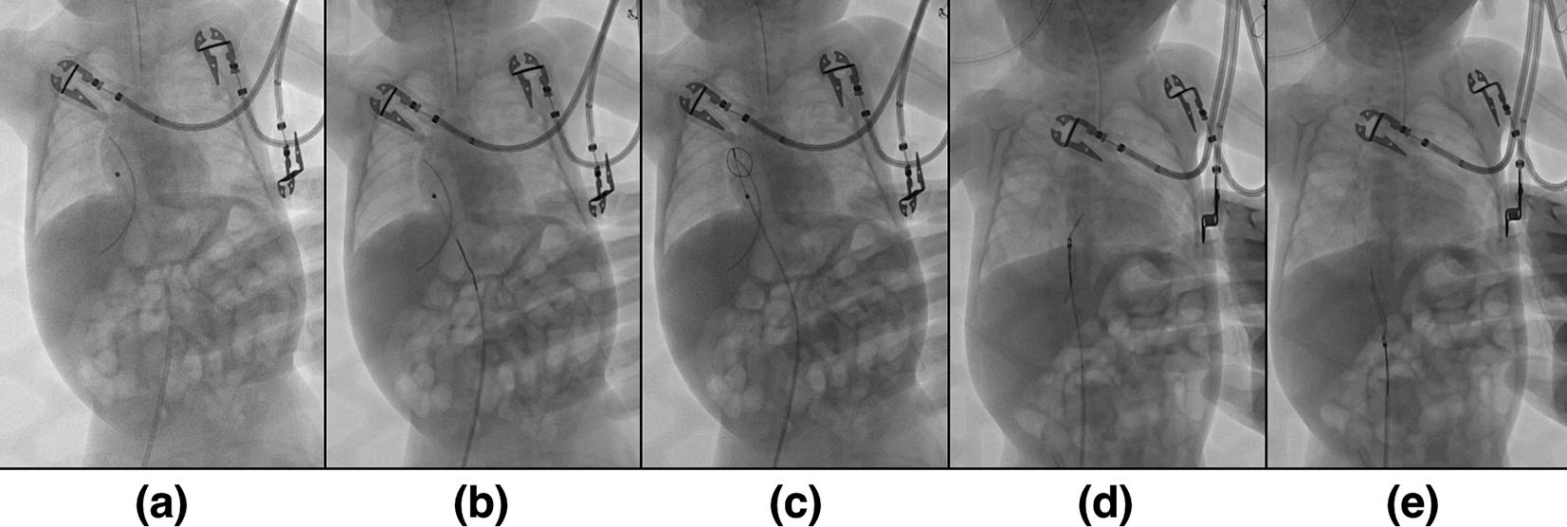
The process of intravascular foreign body extraction. (a) Insertion of guide wire. (b) Insertion of One Snare wire (c). Encirclement of the foreign body with looped wire. (d) Extraction of the foreign body. (e) The foreign body was carried along to the puncture site.

During the procedure, an aseptic and antiseptic action was performed in the right femoral region, where attempts were made to puncture the vein using a 21 Gauge (G) needle under ultrasound guidance. After several unsuccessful attempts at venous puncture, to expedite the process for the patient, a venous incision was performed by the paediatric surgeon team. Following exposure of the femoral vein, puncture was successfully achieved with a 21 Gauge needle, followed by the insertion of a 0.032” guide wire (Terumo ^®^, Tokyo) under low-dose fluoroscopy in the cath lab. Subsequently, a 4 French (Fr) introducer sheath and a 4 Fr vertebra catheter (Terumo ^®^, Tokyo), along with a longer guide wire, were threaded through the sheath and directed towards the inferior vena cava. With precision, an exchange was made using the catheter and the 10 mm, 4 Fr One Snare wire (Merit Medical ^®^, Ireland), which were manoeuvred to encircle the foreign body with its looped wire. Once securely ensnared, the foreign body (the umbilical catheter) was gently extracted. The instruments were subsequently removed, completing the procedure successfully ( Figure 2).

**Figure 3.**
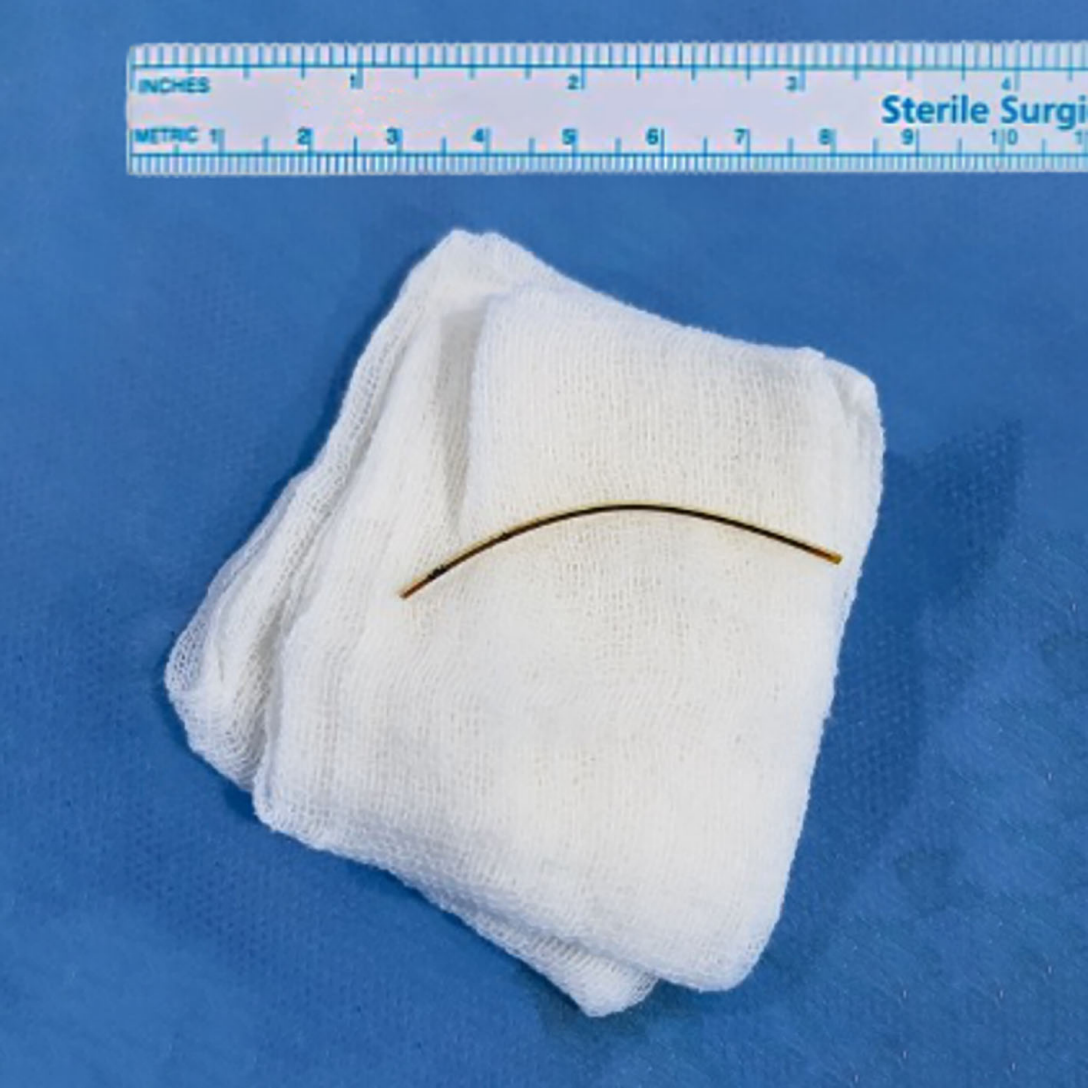
The extracted 5 cm long umbilical catheter.

After the surgery, a 5 cm long umbilical catheter was extracted from the inferior vena cava of the patient ( Figure 3). Two days following the procedure, his clinical condition had improved. The patient had switched to nasal canula on the second postoperative day and had no supplementary oxygen on the third postoperative day. Oral intake was started on the third postoperative day, which was well-tolerated by the patient.

## Discussion

IFB is one of the most severe iatrogenic complications of catheterization. ^2^^,^^3^ It is especially dangerous in neonates who have delicate anatomical structures and limited physiologic reserves. ^2^^,^^4^ However, the umbilical vein catheters are widely used in critical care of the neonates for rapid and dependable central access in preterm babies. Other than being displaced and left behind, injury to other organ during insertion, infection, thromboembolism, fracture of the catheter causing embolization, and even cardiac perforation are notable complications of umbilical vein catheters. ^1^

The case presented herein exemplifies the challenges and complexities associated with IFB retrieval in a severely preterm neonate. Traditional surgical interventions, as attempted initially, proved to be unfruitful for the removal of the IFB in this case. This highlights the anatomical and physiological constraints that traditional surgical methods encounter in such vulnerable patients.

Our experience underscores the utility of an endovascular approach as an alternative, especially when conventional surgical techniques fail or are not feasible. Notably, the minimally invasive nature of the procedure, guided by real-time imaging, allowed for a targeted intervention that minimized tissue disruption and potential complications. Similar imaging technique (low dose fluoroscopy) has been used by some studies about intravascular foreign body retrieval in neonates. ^2^ Given the severely preterm neonate’s frail constitution and the high risks associated with invasive surgical procedures, the endovascular approach offered a less traumatic and more controlled methodology for IFB retrieval.

There are several techniques in performing endovascular extraction of intravascular foreign bodies, such as gripping the foreign body from outside using a snare or forceps, displacing the foreign bodies using balloons or stone retrieval baskets, or using a guidewire and low-profile balloons to grasp the foreign body. ^2^^,^^6^ However, gripping the foreign body is only feasible in relatively large vascular channel, not in smaller vessels such as in neonatal cases. ^2^ Moreover, there are a few purpose-designed devices for intravascular foreign bodies retrieval, such as Amplatz gooseneck (ev3), Trefoiel En-Snare (Merit Medical), Dormia baskets, Alligator retrieval forceps (Cook Medical and ev3), and Myocardial biopsy forceps (Cook Medical). ^3^ These devices can have a range of sizes, variable range of emergences (from 0 degree to 90 degree) and more than one loops, greatly enhancing the manipulation techniques of the IFB retrieval. ^6^ In this case, there are several unique technical challenges, such as difficulty in venous puncture, which requires venous incision by paediatric surgeon team and the use of specialized equipment like a 4 Fr vertebra catheter and the One Snare wire. This technique is also explained by Rossi *et al.* (2018) using the name of “ *Loop snare grasp-guide wire technique*” *.* ^6^ The manipulation of these instruments is crucial and should be undertaken by healthcare providers with expertise in endovascular techniques, especially in such a delicate patient population.

The marked improvement in the patient’s condition post-procedure emphasizes the effectiveness of this approach. Successful removal of the umbilical catheter led to clinical improvements including normalized leukocyte counts and better tolerance to oral intake. Similar results was found after the extraction of embolized umbilical catheter in preterm neonates in other studies. ^1^^,^^2^ Furthermore, the immediate postoperative course, with a quick switch from ventilator support to nasal cannula and eventually to room air, suggests shorter recovery time and lower morbidity, corroborating the benefits of a minimally invasive approach.

The successful removal of an intravascular foreign body *via* endovascular technique in a severely preterm neonate highlights the potential of this approach as a viable alternative to more invasive surgical procedures. However, it is imperative to recognize the technical nuances and challenges involved, especially in this fragile patient population. This case serves not only as a testament to the evolving capabilities of minimally invasive techniques but also as an invitation for further research to optimize and standardize such approaches in neonatal care.

## Consent

Written informed consent for publication of their clinical details and clinical images was obtained from the guardian of the patient.

## Data Availability

All data underlying the results are available as part of the article and no additional source data are required.

## References

[1] Patel J Ramarao S Desai J : A case report of embolized umbilical venous catheter retrieval from the heart via femoral access in 660 g premature neonate. *Radiol Case Rep.* 2019;14:1415–1419. 10.1016/j.radcr.2019.09.006 31700556 PMC6823817

[2] Yadav MK Pillai R Unni M : Iatrogenic Intravascular Foreign Body Retrieval in a Neonate. *J. Clin. Interv. Radiol. ISVIR.* 2020;4:130–132. 10.1055/s-0040-1710151

[3] Woodhouse JB Uberoi R : Techniques for intravascular foreign body retrieval. *Cardiovasc. Intervent. Radiol.* 2013;36:888–897. 10.1007/s00270-012-0488-8 23073559

[4] Pazinato LV Leite TF d O Bortolini E : Percutaneous retrieval of intravascular foreign body in children: a case series and review. *Acta Radiol.* 2022;63:684–691. 10.1177/02841851211006904 33832338

[5] Riley DS Barber MS Kienle GS : CARE guidelines for case reports: explanation and elaboration document. *J. Clin. Epidemiol.* 2017;89:218–235. 10.1016/j.jclinepi.2017.04.026 28529185

[6] Rossi UG Rollandi GA Ierardi AM : Materials and techniques for percutaneous retrieval of intravascular foreign bodies. *J. Vasc. Access.* 2018;20:87–94. 10.1177/1129729818785051 29976095

