# Supplementary material for: Case Report: Endovascular technique for intravascular foreign body retrieval in severely preterm neonate
Source: F1000Res. 2024 Jan 11;13:55. [Version 1] doi: 10.12688/f1000research.142275.1 (PMC11803440; doi:10.12688/f1000research.142275.1)
Supplement: Peer review history

# Reviewer report: Reviewer response for version 1

**DOI:** 10.5256/f1000research.155809.r349487

**Published:** 2024-12-26

**Authors:** Naveen Jain

**Affiliations:** 1 neonatology, KIMSHEALTH Trivandrum (Ringgold ID: 76137), Thiruvananthapuram, Kerala, India; 2 Kerala Institute of Medical Sciences, Trivandrum, Kerala, India

- Important and relevant information. 
- Well written article. 
- Alll details of the case and management details are clearly elucidated, will help many similar high risk babies, an abdominal surgery may be avoided if such information is known to neonatologist . 
- We have reported a similar case of a term born baby (yadav et al; included in the references) 
- The article can be indexed.

Are enough details provided of any physical examination and diagnostic tests, treatment given and outcomes?

Yes

Is the case presented with sufficient detail to be useful for other practitioners?

Yes

Is sufficient discussion included of the importance of the findings and their relevance to future understanding of disease processes, diagnosis or treatment?

Yes

Is the background of the case’s history and progression described in sufficient detail?

Yes

Reviewer Expertise:

neonatology

I confirm that I have read this submission and believe that I have an appropriate level of expertise to confirm that it is of an acceptable scientific standard.

# Reviewer report: Reviewer response for version 1

**DOI:** 10.5256/f1000research.155809.r267553

**Published:** 2024-05-17

**Authors:** Tapas Ranjan Behera

**Affiliations:** 1 Cleveland Clinic, Cleveland, Ohio, USA

Strengths & weaknesses: Unique report regarding a complex case of entrapment of umbilical catheter fragment in a pre-term neonate that was successfully managed by endovascular retrieval of the foreign body. Elaboration and discussion about the possible causes of foreign body entrapment in this case would make the report informative.

 Short summary & relevance: Intravascular foreign body retrieval in neonates are critical to deal with, more so when the neonate is pre-term. The authors have described the effective removal of the foreign body by the endovascular approach.

Are enough details provided of any physical examination and diagnostic tests, treatment given and outcomes?

Yes

Is the case presented with sufficient detail to be useful for other practitioners?

Yes

Is sufficient discussion included of the importance of the findings and their relevance to future understanding of disease processes, diagnosis or treatment?

Yes

Is the background of the case’s history and progression described in sufficient detail?

Yes

Reviewer Expertise:

Surgical improvisation, Cancer Therapy. Past experience with similar case of lodged peritoneal dialysis catheter which had lead to a published case report.

I confirm that I have read this submission and believe that I have an appropriate level of expertise to confirm that it is of an acceptable scientific standard.
